# Psychoeducational Intervention for Caregivers of Adolescents and Young Adults with Psychiatric Disorders: A 7-Year Systematic Review

**DOI:** 10.3390/jcm13237010

**Published:** 2024-11-21

**Authors:** Rosaria Di Lorenzo, Alice Dardi, Valentina Serafini, Mei Joy Amorado, Paola Ferri, Tommaso Filippini

**Affiliations:** 1 Mental Health Department and Drug Abuse, AUSL-Modena, 41121 Modena, Italy; 2 School of Specialization in Psychiatry, University of Modena and Reggio Emilia, 41125 Modena, Italy; alice.dardi@gmail.com (A.D.); valeserafini90@gmail.com (V.S.); 3 Nursing Programme, University of Modena and Reggio Emilia, 41125 Modena, Italy; 271672@studenti.unimore.it; 4 Department of Biomedical, Metabolic and Neural Sciences, University of Modena and Reggio Emilia, 41125 Modena, Italy; paola.ferri@unimore.it; 5 Environmental, Genetic and Nutritional Epidemiology Research Center (CREAGEN), Section of Public Health, Department of Biomedical, Metabolic and Neural Sciences, University of Modena and Reggio Emilia, 41125 Modena, Italy; tommaso.filippini@unimore.it; 6 School of Public Health, University of California Berkeley, Berkeley, CA 94704, USA

**Keywords:** caregiver burden, caring experience, quality of life

## Abstract

**Background/Objectives:** Psychoeducation is a specialized form of psychological education aimed at helping people suffering from psychological problems and their families. To investigate the efficacy of psychoeducation interventions (PEIs) in improving both the burden and quality of life of caregivers and the health conditions of their adolescent or youth patients. **Methods:** The following databases were used: PubMed, PsycInfo, CINAHL Plus with full text, Medline and Nursing Reference Center Plus. Two search strings were developed, one for the mental health conditions of assisted patients and the other one for caregivers. **Results:** We selected 30 articles and applied two differentiated meta-analyses on 12 of them to evaluate the effectiveness of PEIs. We highlighted a statistically significant superior efficacy of PIEs compared to control groups in five studies in the meta-analysis of studies on caregiver outcomes, and eight studies in the meta-analysis of studies on outcomes of patients cared for. **Conclusions:** PEIs were shown to be effective in reducing symptoms and hospitalizations in persons cared for, improving their quality of life as well as that of their caregivers. Regarding the caregiver’s care burden, our review suggests that PEIs generally improve burden in caregivers, reducing the perception of their workload in caring for adolescent or youth persons.

## 1. Introduction

### 1.1. Psychoeducation

Family psychoeducation is a support and information service provided by mental health professionals. It is specifically designed to deliver accurate information about mental illness to psychiatric patients and their caregivers, with the aim of enhancing their understanding of mental illness and enabling more effective management of its consequences [1]. According to recent clinical practice guidelines [2], psychoeducation combines methods and concepts based on cognitive behavioral therapy, group therapy and education and consists of four essential activities: 1. Informing patients about their illness; 2. Problem-solving training; 3. Communication training; 4. Self-affirmation training. Family psychoeducation is one of the types of psychoeducation based on the target population, individual, family, group or community.

This specialized form of education aims to help individuals suffering from psychological problems, as well as their families, to better understand the nature of their disorders and provide useful tools for managing them. It addresses the need for those dealing with such disorders to gain more information about symptoms, causes, medication side effects, and techniques to intervene and reduce complications. In this way, it provides a sense of understanding and control over the situation. The program includes various activities, such as assessing family resources and challenges, setting goals for each family member and the family as a whole, providing in-depth information about the psychiatric disorder and its treatment, teaching communication skills and structured problem-solving methods, and using specific strategies to handle critical situations like suicide risk or non-compliance with medication. Moreover, it involves integrating psychotherapeutic or psychoeducational treatments with rehabilitative and pharmacological treatments to maximize benefits for the patient. Another crucial aspect is the early identification of crisis signs to allow timely intervention and prevent the worsening of disorders. Its primary purpose is to enhance the quality and frequency of family communication, which in turn leads to improved interpersonal relationships and therapeutic outcomes [3].

Systematic reviews of controlled studies on family interventions for patients with early psychosis have shown that psychosocial interventions, such as cognitive behavioral therapy and psychoeducation programs, can significantly improve the psychosocial functioning of families and their knowledge about the illness and its treatment. However, these interventions may have only small or non-significant effects on other family outcomes, such as caregiving burden and long-term positive experiences or benefits. Despite the psychological support and resources provided by health professionals, some families struggle to regularly attend psychoeducation sessions due to time constraints, a sense of social or internal stigma related to mental illness, and negative experiences in seeking help. Hence the need for self-help programs that can empower patients with chronic mental and physical illnesses (such as depression, anxiety, and eating disorders) and their caregivers has emerged [4]. Generally, psychoeducational techniques are used in combination with a wide range of psychotherapeutic (cognitive behavioral, systemic relational, psychodynamic, group and others), rehabilitative, psychosocial and pharmacological treatments for managing psychosis [5]. Previous studies have shown that these interventions can be effective in preventing disease relapse and reducing hospitalization rates [6]. Several quantitative studies have highlighted that group psychoeducation programs can increase patients’ awareness of relapse characteristics, improve treatment adherence and enhance quality of life. However, few studies have been conducted in inpatient psychiatric settings, where patients typically have more severe impairments than outpatients, and most patients experience manic or psychotic episodes which require hospitalization. It remains unclear whether psychoeducational programs can improve routine psychiatric care for severely ill patients due to the high rates of dropout in psychoeducational interventions, especially in long-term therapeutic programs [7]. Some authors [8] have emphasized that, during the acute phase of psychosis, treatments should start as early as possible because in these conditions, therapists can get to know the unconscious world of the patient, helping him/her to better understand their feelings and needs. In any case, the early intervention paradigm in psychotic disorders represents a well-defined and effective therapeutic approach that addresses the early manifestation of psychosis. The term “early intervention in psychosis” (EIP) implies the hypothesis that the longer the duration of untreated psychosis (DUP) the worse the outcome [9]. EIP provides multidisciplinary care and practical preventive strategies, promotes recovery and increases access to care [10].

A recent study, which explored which aspects of mental health and social care services people with first-episode psychosis (schizophrenia and bipolar spectrum disorders) considered important for their long-term recovery, found that services played an indirect role in long-term recovery by supporting patients’ personal resources, therefore recommending better coordination of services [11].

### 1.2. Caregivers

Family members caring for a young person with a psychiatric diagnosis face high levels of stress, depression, and social isolation. Despite evidence of their effectiveness, accessing specialized family interventions remains a significant challenge. The presence of close family and friends supporting a loved one with psychosis is crucial for promoting a better prognosis and well-being. However, the responsibility of care can be very burdensome, making caregivers vulnerable to physical and mental illnesses. For this reason, they need access to psychosocial treatments that include the knowledge and support necessary to care for their loved ones and maintain their own well-being [12]. Family caregivers may have various emotional reactions, but common themes include guilt, increased anxiety, and, in the case of prolonged recovery, a sense of loss. Mood and psychotic disorders, along with their treatments, are known to be associated with high levels of stress, distress, and depression among caregivers. In summary, family members face significant mental health challenges. This can create tensions among family members, and siblings may suffer from a reduced quality of life as parents need to invest more in caring for their relatives [13]. The high levels of stress and family conflict often faced by caregivers can negatively impact the care and overall well-being of the patient. Specifically, caregivers with high caregiving burdens and lacking effective coping strategies and resources may exhibit intense emotions toward the patient, increasing the risk of impairment. Therefore, family-oriented interventions that include support and training elements are crucial for meeting the health needs and caregiving roles of caregivers, especially when caring for a young relative with psychosis for the first time [4].

Research shows that family caregivers can participate in and benefit from programs led by other caregivers [14]. This intervention model provides evidence that peer-led mental healthcare initiatives should be further integrated, developed and studied. However, the roles of experienced caregivers require not only support and supervision practices, but also financial investment to ensure these roles are supported and compensated, offering meaningful opportunities for people with caregiving experience to share their knowledge and provide support to others with similar experiences [14]. Other research supports the idea that psychoeducational interventions led by people with mental health problems, i.e., expert users, lead to positive outcomes, such as improved mental well-being, reduced burden, and enhanced empowerment. These findings indicate that peer support opportunities should be encouraged and supported. In particular, programs developed and implemented by experienced caregivers are well-received by caregivers of people with mental health difficulties.

Moreover, the request for psychoeducational components and the development of additional skills were welcomed, as was the guidance of this process by caregivers, and this innovative program showed promising results [14].

## 2. Materials and Methods

### 2.1. Study Design

This systematic review aims to investigate the meaning and effectiveness of psychoeducation in the context of mental health, with a particular focus on its impacts on the caregivers or family members of adolescents or young adults with psychiatric illnesses.

This review was conducted in accordance with the PRISMA guidelines [15]. The protocol was registered with PROSPERO (registration no. CRD42023429802), the international prospective register of systematic reviews, to ensure methodological transparency and to prevent duplication of research efforts.

### 2.2. Research Questions

The research questions are the following:

1. What is psychoeducation in the context of caring for adolescent patients with psychiatric illness, in which settings is it used, and who administers it?
2. Does psychoeducation directed at caregivers of adolescent and young adult patients with psychiatric illness affect the prognostic outcomes of the illness? And what effects are observed on the caregivers?
3. How and by whom is the psychoeducational setting applied? How are the outcomes measured?
4. What is the optimal timing for an effective psychoeducational intervention for caregivers of adolescent patients with psychiatric illness, and what should be the follow-up period to verify its outcomes?

### 2.3. Eligibility Criteria

#### 2.3.1. Inclusion Criteria

- Intervention: Studies that examined psychoeducational interventions designed to support caregivers of adolescent and young adult patients with psychiatric illness.
- Population: Studies involving caregivers of adolescents and young adults (aged 11–25) diagnosed with any psychiatric illness.
- Publication Date: Articles published from 2017 to 2024, in order to investigate the most recent research on this topic.
- Study Design: Randomized controlled trials (RCTs) and observational qualitative studies.
- Language: Studies published in the Italian or English languages.

#### 2.3.2. Exclusion Criteria

- Case reports, editorials, commentaries, and review articles.
- Studies not providing specific data on adolescents and young adults (age > 25 years old).
- No English or Italian language.
- Publication date before 2017.

### 2.4. Search Strategy

A comprehensive search strategy was developed to identify relevant studies from multiple electronic databases, including PUBMED, CINAHL PLUS WITH FULL TEXT, APA PSYCINFO, MEDLINE, and NURSING REFERENCE CENTER PLUS. The search was conducted in 2021 and 2023. For the literature search, two search strings were used, related to each other through the use of Boolean operators:

- (Psychoeducation) AND (“Psychiatric Disorders” OR “Psychosis” OR “Mental Illness”) AND (“adolescents” OR “young adults”).
- (Psychiatric Disorders) AND (“Caregivers” OR “Family Members”).

### 2.5. Study Selection

By applying the specific selection criteria described, a total of 1195 articles were identified. The entire bibliography was downloaded and imported into Zotero 6, software for managing bibliographic references and related materials (e.g., PDF files), to facilitate subsequent screening phases. From the initial articles, 459 duplicates were removed, resulting in 736 studies.

**Figure 1:**
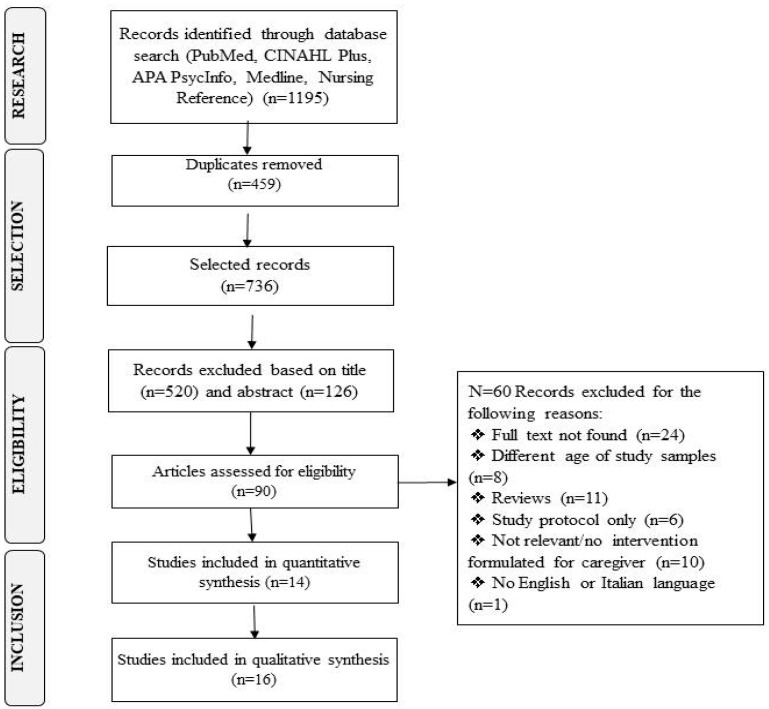
Flowchart of study selection.

With these premises, an exclusion-based screening activity began, initially, based on the titles, excluding 520 non-relevant articles, and secondly, based on the article abstracts, excluding 126 studies. Thus, a total of 90 articles emerged for in-depth review, which were further revised by eliminating articles without full texts available. Following screening, 66 remaining articles were read and analysed in their entirety. From these, eight articles with a sample of ages different from the inclusion criteria were excluded, 11 reviews, six studies on research protocols, 10 studies without caregiver interventions, and one due to the language being neither Italian nor English. The process and reasons for the inclusion and exclusion of the remaining documents are presented in the flow-chart in Figure 1.

### 2.6. Data Extraction

The selected studies perfectly reflect the established criteria, from which data of interest were extrapolated, namely general information about the publication, the setting and study methodology, sample data (size, female and male percentages, age range, type of psychiatric illness and type of intervention on the caregiver and/or the patient), and the main outcomes that address the research questions listed above. These elements were entered into a custom-made Excel database to allow for quantitative and qualitative analysis of the collected data.

### 2.7. Quality Assessment

The methodological quality of the included studies was assessed using the Revised Cochrane Risk of Bias Tool for RCTs (RoB 2). The quality assessment was performed independently by two reviewers, and any disagreements were resolved by consensus or by consulting a third reviewer.

### 2.8. Data Synthesis

A narrative synthesis was conducted due to the heterogeneity of the included studies in terms of interventions and outcomes. Where possible, quantitative data were pooled in a meta-analysis using a random effects model to account for variability among studies. Statistical heterogeneity was assessed using the I² statistic, with values above 50% indicating substantial heterogeneity. We performed a meta-analysis comparing scores at the end of the intervention versus baseline in both treatment and control groups. We did this through computation of Hedges’ g standardized mean differences (SMDs), due to the heterogeneity in the measurements of outcomes using a DerSimonian–Laird random effects model with the ‘meta’ routine of the Stata statistical software (Stata 18.0-MP 2023, StataCorp LLC, College Station, TX, USA).

### 2.9. Ethical Considerations

As this study was a systematic review of published literature, ethical approval was not required. However, the review process was conducted with rigorous adherence to ethical standards in research, including transparency, accuracy and avoidance of bias.

## 3. Results

### 3.1. Characteristics of the Studies

At the end of the selection process described in Figure 1, we selected 30 studies that met the research questions and inclusion criteria. The characteristics of the selected studies are presented in Table 1.

The selected studies were conducted in geographic areas covering all continents. Specifically, four studies were from Europe, including three from the United Kingdom [12,16,17] and one from Spain [18]. Another nine studies were from Asia, with three from China [19,20] (including one from Hong Kong [21]), two from Japan [4,22], two from Indonesia [23,24], one from India [1] and one from Singapore [25]. Additionally, there was one study from Egypt [26] and one study conducted in Australia [27]. Finally, the remaining studies were from the Americas, with one from Puerto Rico [28], two from Canada [29,30] and 12 from the USA [31,32,33,34,35,36,37,38,39,40,41,42]. We observed a clearly greater prevalence of studies from the USA and note that no study was conducted in Italy.

The inclusion criteria time window for the studies covered a period between 2017 and 2023. It can be observed that the most recent studies, published between 2021 and 2023, totaled nine [12,18,19,20,21,22,28,30].

The follow-up periods of the studies ranged from 2 months [24] to 4 years [19,30,34], whereas two studies did not specify any follow-up period [27,29]. The average follow-up duration of all studies was 16.22 months ± 17.95 SD.

In 21 of the included studies, the design was randomized controlled trials [1,4,16,17,19,21,22,23,24,26,28,32,33,34,35,36,37,38,40,41,42].

The setting in which the examined studies were conducted was predominantly outpatient or non-hospital in all studies but two where the setting was a hospital environment [27,31].

In analyzing the outcomes of psychoeducation, nine of the selected studies assessed the outcomes only in caregivers (Table 2), another seven studies only in patients receiving care (Table 3), and the remaining 14 studies (Table 4) in both caregivers and patients.

**Table 1:** Characteristics of selected studies.

N	Authors, Year of Publication, Country	Study Design	Caregivers		Patients			
			No.	Type	N.	Female %	Age (Range or Mean ± SD)	Disorders
1	Batchelor et al., 2022 [12], UK	Qualitative	35	Parents	NA	NA	11–21	Schizophrenia
2	Beck et al., 2020 [16], UK	RCT	NA	NA	112	99.1	15.8 ± 1.1	Borderline personality disorder
3	Bernal et al., 2019 [28], Puerto Rico	RCT	NA	Parents	121	53.4	13–17.5	Major depressive disorder
4	Chien et al., 2018 [19], China	RCT	210	Parents	210	NA	21–44	Psychotic onset
5	Chien et al., 2020 [4], Japan	RCT with three groups	114	Parents	114	NA	G1: 24.2 ± 6.8 G2: 26.2 ± 7.8 G3: 26.5 ± 7.8	Psychotic onset
6	Izon et al., 2020 [17], UK	Qualitative	14	Mothers	14	36	17–34	Psychosis
7	Katsuki et al., 2018 [22], Japan	RCT	49	Parents	49	49	18–85	Major depressive disorder
8	Kopelovich et al., 2021 [31], USA	Qualitative	29	Parents	NA	NA	15–86	Schizophrenic spectrum
9	Lal et al., 2019 [29], Canada	Qualitative	24	Mothers	NA	NA	15–24	Prodromes of psychosis
10	Lo et al., 2022 [20], China	Qualitative	13	Parents	13	20	15–30	Psychosis
11	Marchira et al., 2019 [23], Indonesia	RCT	50	Parents	50	39	22.4 ± 4.5	Psychosis
12	Miklowitz et al., 2020 [32], USA	RCT	NA	Mothers	127	60.7 (FFT) 68.2 (EC)	9–17	Major depressive disorder, bipolar disorder
13	Miklowitz et al., 2021 [33], USA	RCT	25	Parents	34	44.1	13–25	Mood disorders, Psychosis
14	Miklowitz et al., 2022 [34], USA	RCT	114	Parents	114	64	9–17	Major depressive disorder, bipolar disorder
15	Nolan and Petrakis, 2019 [27], Australia	Qualitative	NA	Parents	NA	NA	16–64	Psychotic onset
16	O’Donnell et al., 2017 [35], USA	RCT	70	Parents	141	50.0 (FFT) 60.6 (EC)	15.6 ± 1.4	Bipolar disorder
17	O’Donnell et al., 2020 [36], USA	RCT	70	Parents	144	50.0 (FFT) 59.7 (EC)	15.6 ± 1.4	Bipolar disorder
18	Peris et al., 2017 [37], USA	RCT	NA	Parents	62	44	8–17	Obsessive compulsive disorder
19	Perlick et al., 2018 [38], USA	RCT	43	Parents	40	62.5	34.2 ± 14.8	Bipolar disorder
20	Pollio et al., 2017 [39], USA	Qualitative	123	Parents	123	50	35 ± 14	Schizophrenia, Bipolar disorder, Major depressive disorder
21	Rahayu et al., 2019 [24], Indonesia	RCT	11	Orphanage operators	77	42.9	Mean: 14	Prodromes of psychosis
22	Rami et al., 2018 [26], Egypt	RCT	60	Family members	60	30	23–46	Schizophrenia
23	Rinne et al., 2021 [40], USA	RCT	105	Parents	58	39.1	<19	Psychosis
24	Sepúlveda et al., 2019 [18], Spain	RCT	53	Family members	40	90.5	23.9 ± 6	Eating disorder
25	Sheikhan et al., 2021 [30], Canada	Qualitative	13	Family members	13	53.8	14–18	Generic mental disorders
26	Verma et al., 2019 [1], India	RCT	30	Family members	30	0	<30–>36	Schizophrenia
27	Weintraub et al., 2019 [41], USA	RCT	NA	NA	145	54.5	12–18	Bipolar disorder
28	Weintraub et al., 2021 [42], USA	RCT	203	Mothers	127	66.9	9–17	Mood disorders
29	Wong et al., 2019 [25], Singapore	Qualitative	19	Parents	49	57.4	16–40	Psychosis
30	Zhang et al., 2023 [21], Hong Kong	RCT	65	Cohabitants	18	50	<35	First psychotic episode

EC—Enhanced care; FFT—family-focused therapy; NA—not available; RCT—randomized controlled trial; SD—Standard Deviation.

### 3.2. Characteristics of the Sample

The characteristics of the samples analyzed in the included studies are reported in Table 1. As established by the inclusion criteria, studies were considered eligible if the patients were adolescents or young adults. It was observed that, in some cases, the patients’ ages were recorded as a range, while in others, as an average. During the data analysis and extraction phase, the patients’ diagnoses were also considered. It was found that in the majority of studies [n = 16], the patients suffered from schizophrenia spectrum disorders [1,4,12,17,19,21,23,24,25,26,27,29,31,33,39,40], whereas the following patient disorders were less frequently reported: mood disorders in 11 studies [22,28,32,33,34,35,36,38,39,41,42], including bipolar disorder; eating disorders [18], borderline personality disorder [16] and obsessive-compulsive disorder [37]. Only one study reported generically “mental disorders” [30].

It should be noted that, although there were 30 studies, the samples analyzed by the studies are 29, as two articles [35,36] refer to the same sample, with the second study analyzing the follow-up of the first. As seen in Table 3 and Table 4, the percentage of female patients was predominant in 10 studies, reaching 99.1% in the study by Beck et al. [16]. The sex ratio in the samples is 50% in two studies [21,39] and is less than 50% in 12 other studies. The study by Verma et al. [1] included only male patients, while six other studies did not specify gender data [4,12,19,27,29,31]. It is important to mention that in the studies by Chien et al. [19] and Marchira et al. [23], all demographic data refer to a single sample that includes family units where the characteristics of caregivers versus those of patients are not specified. Another noteworthy finding concerns the type of caregiver. In 22 selected studies, parents were the most represented type; in particular, in four studies the caregiver is the patient’s mother [17,29,32,42]. Of the remaining studies, four referred to family members in general [1,18,26,40], one study to orphanage staff [24], another to cohabitants [21] and two other studies did not specify this information [16,41].

### 3.3. Risk of Bias Assessment

As shown in Table 5, the risk of bias assessed through the Revised Cochrane Risk of Bias Tool for RCTs was low in most of the included studies.

### 3.4. Quantitative Outcomes

More than half of the analyzed articles (20 out of 30) provided quantitative outcomes. All the selected studies assessed the outcomes of psychoeducational interventions (PEIs) in caregivers (Table 3), patients alone (Table 4), or both (Table 5), using psychometric scale scores which were all different from each other, although outcomes can be grouped into macro areas of investigation.

### 3.5. Outcomes in Caregivers

More than Stress (“Burden”, “Stress/Psychological Health”), Caregiving Experience (“knowledge of the disorder”), and Family Functioning (“Conflicts” and “Cohesion”) are the principal areas analyzed by the studies focused on caregiver outcomes. These articles evaluated potential correlations between PEIs in caregivers and changes in scale scores:

1. First item Chien et al. [19]: Reduction in “Burden” on the FBIS (Family Burden Interview Scale) at the end of the post-treatment psychoeducational follow-up, with scores from 30.98 ± 6.45 SD to 27.01 ± 8.92 SD. Additionally, an improvement in family functioning on the FAD (Family Assessment Device) was observed at the end of the post-treatment follow-up, with scores from 22.93 ± 7.32 SD to 26.02 ± 12.89 SD in the treatment group, compared to changes from 24.88 ± 8.72 SD to 23.12 ± 10.23 SD in the control group.
2. Second item Chien et al. [4]: Improvement in “Burden” on the FBIS with scores from 29.92 ± 5.01 SD to 26.13 ± 7.12 SD, and in caregiving experience on the ECI (Experience of Caregiving Inventory) scale, with scores from 133.22 ± 16.52 SD to 119.53 ± 18.81 SD in the group treated with family psychoeducation, compared to scores from 133.02 ± 18.42 SD to 141.81 ± 19.21 SD in the control group. Additionally, an improvement in social problem-solving ability was noted on the SPSI-R (Social Problem-Solving Inventory-Revised) with scores from 50.23 ± 7.03 SD to 50.82 ± 9.05 SD.
3. Katsuki et al. [22]: Reduction in psychological stress on the K6 (Kessler Screening Scale for Psychological Distress) with scores from 5.2 ± 3.3 to 4.82 ± 4.056 SD in the group treated with brief multi-family psychoeducation, compared to changes from 5.6 ± 4.4 SD to 4.34 ± 3.72 SD in the control group.
4. Marchira et al. [23]: Improvement in caregivers’ knowledge of psychosis treated with brief psychoeducation, as measured by the KOP (Knowledge of Psychosis) scale, with scores in the treatment group from 5.78 ± 1.92 SD to 10.08 ± 2.77 SD at follow-up, compared to scores from 5.36 ± 1.94 SD to 4.56 ± 1.83 SD in the control group.
5. Miklowitz et al. [33]: Decrease in family conflicts as assessed by the CBQ (Conflict Behavior Questionnaire) in the group treated with high–low intensity family-focused therapy training, with scores in the treatment group changing from baseline 10.0 ± 6.1 SD to outcome 7.8 ± 5.3 SD vs. control group from 8.6 ± 7.2 SD to 5.5 ± 6.4 SD.
6. Peris et al. [37]: Reduction in conflicts on the FES (Family Empowerment Scale): −1.26 in the group treated with positive family interaction therapy vs. +0.05 in the control group, and improvement in family cohesion also on the FES scale: +0.60 in the treated group vs. −0.23 in the control group. A reduction in the score on the FAS (Family Accommodation Scale) was also observed in the family members: −17.02 in the treated group vs. −7.48 in the control group.
7. Perlick et al. [38]: Improvement in the overall psychological health of caregivers treated with the family-focused psychoeducational intervention on the SF-MCS (SF-36 Mental Component Summary) with an improvement percentage of 41% in the treated group vs. 21% in the control group and a reduction in depression indices (CES-D, 48% in the treated group vs. 22% in the control group).
8. Sepúlveda et al. [18]: Reduction in negative reactions to illness as measured by the FQ (Family Questionnaire). The treated group went from baseline scores of 20.77 ± 6.26 SD to follow-up scores of 21.12 ± 5.65 SD, while the control group went from 22.96 ± 4.87 SD to 21.5 ± 5.04 SD. Improvement in symptom acceptance indices on the AESED scale (Accommodation and Enabling Scale for Eating Disorders), in both groups. Furthermore, an improvement in emotional well-being indices and caregivers’ awareness of their resources was observed on the GHQ-12 (General Health Questionnaire), HADS (Hospital Anxiety and Depression Scale), ECI (Experience of Caregiving Inventory), EDSIS (Eating Disorder Symptom Impact Scale) and Brief-IPQ (Brief Illness Perception Questionnaire) in both groups.
9. Verma et al. [1]: Increase in knowledge and understanding of the illness by caregivers treated with the psychoeducational intervention. Improvement in quality of life indicators on the BREF (Quality of Life-Bref scale): the treated group went from baseline scores of 36.47 ± 5.82 SD to an outcome of 51.87 ± 6.67 SD, while the control group went from 36.47 ± 5.82 SD to 32.27 ± 5.06 SD.
10. Zhang et al. [21]: Slight worsening of the primary outcome score “Caregivers Burden” as assessed by the ZBI (The Zarit Burden Interview). The treated group went from baseline scores of 39.52 ± 13.83 SD to follow-up scores of 39.70 ± 15.31 SD, while the control group went from 42.09 ± 16.94 SD to 40.81 ± 15.01 SD. However, a significant improvement was observed in the secondary outcome on the Family Impact subscale (a subcategory of the ECI—Experience of Caregiving Inventory test).

In addition, three articles, two by Weintraub et al. [41,42] and one by Miklowitz et al. [32], also contributed to caregiver outcomes, but they were not included in the meta-analysis as they reported cryptic numerical data that could not be used.

- Miklowitz et al. [32]: Reduced vulnerability to bipolar disorder evaluated on the FFT scale.
- Weintraub et al. [41]: Reduction in family conflict indices on the CBQ (Conflict Behavior Questionnaire) in families of patients with comorbidity between bipolar disorder and ADHD who underwent psychoeducational treatment.
- Weintraub et al. [42]: Maternal stress levels on the SCL-9 (Symptom Checklist-90 Revised) decreased by an average of 0.41 at each 4-month follow-up. Psychoeducational treatment improved family cohesion levels on the FACES-II (Family Adaptability and Cohesion Scale-II) and, consequently, maternal stress levels in the long term.

### 3.6. Outcomes in Patients Cared for by Caregivers

Regarding patient outcomes, 12 studies analyzed changes in symptom scale scores of patients cared for by caregivers treated with PEIs. These studies investigated patient outcomes concerning the following disorders:

#### 3.6.1. Borderline Personality Disorder (BPD)

- Beck et al. [16]: The BPFS-C (Borderline Personality Feature Scale for Children) score showed no statistically significant difference between the group receiving a psychoeducational intervention for caregivers (mentalization-based group therapy) and the control group at the end of follow-up (71.3 ± 15.0 SD in the treated group vs. 71.3 ± 15.2 SD in the control group). Secondary outcomes included various specific symptoms related to borderline disorder, with no statistically significant differences between the groups concerning self-harm (RTSHIA), depression (BDI-Y), externalizing/internalizing symptoms (YSR), and social functioning (CGAS).

#### 3.6.2. Mood Disorders (MD)

- Miklowitz et al. [33]: No significant changes in patient health on the PHQ-9 (Patient Health Questionnaire) from pre- to post-treatment with High–Low Intensity family-focused therapy.
- Perlick et al. [38]: Following psychoeducational treatment (family-focused treatment adapted solely for the caregiver), there was a reduction in depression scores on the HAM-D (Hamilton Depression Rating Scale). The treated group showed a reduction from 15.22 to 5.85 vs. 14.53 to 10.11 in the control group.
- Rinne et al. [40]: Depression scores on the CDS (Calgary Depression Scale) were reduced with treatment (CDS pre-treatment: 5.94 ± 5.33 SD vs. CDS post-treatment 3.23 ± 4.23 SD), regardless of the type of treatment administered (family-centered therapy for the intervention group or psychoeducation for the control group).

#### 3.6.3. Schizophrenia Spectrum Disorders (SSD)

- Chien et al. [19]: Reduction in psychotic symptoms in the PANSS (Positive and Negative Syndrome Scale) at the end of post-treatment follow-up (family support groups), with scores from 97.67 ± 9.98 SD to 75.55 ± 14.38 SD for the treated group, compared to 97.12 ± 10.38 SD to 97.65 ± 19.87 SD for the control group.
- Chien et al. [4]: Reduction in psychotic symptoms on the PANSS at the end of treatment (family psychoeducation). The treated group showed a score change from 107.22 ± 14.71 SD at baseline to 104.11 ± 19.51 SD, while the control group showed a change from 118.12 ± 9.81 SD to 138.82 ± 19.81 SD.
- Marchira et al. [23]: Non-statistically significant reduction in psychotic symptoms on the PANSS at six months post-intervention follow-up (brief psychoeducation). While the control group changed from 78.98 ± 17.73 to 38.90 ± 13.24 SD, the treated group changed from 74.46 ± 15.67 SD to 38.90 ± 13.24 SD.
- Rahayu et al. [24]: Reduction in prodromal psychosis symptoms on the PQ16 (Prodromal Questionnaire-16) for the group treated with cognitive therapy and family psychoeducation, with a change from 9.47 to 6.32 (*p* = 0.00).
- Rami et al. [26]: Statistically significant reduction in psychotic symptoms on the PANSS, with a difference between the group treated with a psychoeducational intervention (behavioral family psychoeducation program) and the control group (t = 7.3; *p* < 0.001).
- Zhang et al. [21]: Better recovery levels recorded on the MHRM in the treated group compared to the control group: Cohen’s d = 1.391 (but did not reach statistical significance).

#### 3.6.4. Eating Disorders [EA]

- Sepúlveda et al. [18]: Improvement in behaviors associated with eating disorders in the group treated with psychoeducation, as measured by the EAT-26 (Eating Attitudes Test-26). The control group showed a change from 27.91 ± 16.06 SD to 15.36 ± 16.96 SD at follow-up vs. the psychoeducation-treated group change from 30.20 ± 14.48 SD to 24.50 ± 12.39 SD (*p* = 0.001) at follow-up.

#### 3.6.5. Obsessive-Compulsive Disorder (OCD)

- Peris et al. [37]: Studied OCD patients, evaluating the outcomes of a psychoeducational intervention for caregivers (positive family interaction therapy) using a non-disorder-specific scale, with better response rates on the Clinical Global Impression-Improvement Scale (68% treated group vs. 40% control group).

Additionally, several articles aggregated outcomes on patients but were excluded from the meta-analysis due to incomplete data:

- Bernal et al. [28]: Psychoeducational treatment (psychological education workshops) did not accelerate the reduction of depressive symptoms on the CDI (Children’s Depression Inventory) scale.
- Miklowitz et al. [32]: Unchanged scores on the Suicide Ideation Questionnaire (SIQ) in both groups—17% treated group vs. 14% control group.
- Miklowitz et al. [34]: Youth with specified BD (vs. major depressive disorder), younger age, earlier symptom onset, more severe mood symptoms, lower psychosocial functioning, and more familial conflict over time had higher mood instability ratings throughout the study period. Mood instability mediated the association between baseline diagnosis and mother/offspring conflict at follow-up. Psychosocial interventions did not moderate these associations. A questionnaire measure of mood instability tracked closely with symptomatic, psychosocial, and family functioning in youth at high risk for BD. Interventions that are successful in reducing mood instability may enhance long-term outcomes among high-risk youth. In a mixed-effects regression model, random assignment to the FFT (family-focused therapy) or control group was not related to total CALS (Children’s Affective Lability Scale) scores. FFT combined with pharmacotherapy was associated with longer periods free from mood episodes and greater reductions in suicidal ideation and behavior among young individuals at high risk for bipolar disorder.
- O’Donnell et al. [35]: Improvements in quality of life on the KINDL in the dimensions of physical well-being and friendship skills at follow-up for the group treated with family-centered treatment for adolescents.
- O’Donnell et al. [36]: Patients in the group treated with family-centered treatment experienced improvements in family cohesion, adaptability, and a reduction in intra-family conflicts.
- Weintraub et al. [41]: Manic symptoms of patients with comorbid bipolar disorder and ADHD showed an 18% reduction in the treated group compared to a 2% reduction in the control group on the PSR (Psychiatric Status Rating Scale).

Lastly, remission rates for psychiatric disorders post-psychoeducational treatment were assessed in three articles:

- Beck et al. [16]: Remission rate of borderline disorder remained unchanged at 29% between the group treated with a psychoeducational intervention for caregivers (mentalization-based group therapy) and the control group.
- Bernal et al. [28]: Remission rate of depression was 70% in both groups at follow-up, regardless of psychoeducational treatment (psychological education workshops).
- Peris et al. [37]: The psychoeducational treatment (positive family interaction therapy) resulted in an increase in OCD remission rates of 58% for the treated group vs. 27% for the control group.

### 3.7. Meta-Analysis

To perform the meta-analysis, the studies were divided into two groups depending on whether the outcomes were assessed in the people receiving care or in the caregivers.

#### 3.7.1. The Meta-Analysis with the Studies Analyzing Outcome in Caregivers [Figure 2]

- it included 10 studies [1,4,18,19,21,22,23,33,37,38];
- the follow-up time in the studies analyzing the outcome on caregivers was 7.5 months ± 13.7 SD in five studies. PEIs obtained a superior efficacy on caregivers’ outcomes in a statistically significant way compared to control groups without PEIs:
  the study by Chien et al. [4], which reported an improvement in burden and in caregiving experience; the study by Marchira et al. [23], which reported an improvement in caregivers’ knowledge of psychosis; the study by Peris et al. [37], which observed a reduction in conflicts on the Family Empowerment Scale; the study by Perlick et al. [38], which reported an improvement in overall psychological health; the study by Verma et al. [1], which showed a statistically significant improvement in quality of life at the end of the follow-up;
- the I^2^ score = 95% indicates the high heterogeneity of the model.

#### 3.7.2. The Meta-Analysis with Studies Analyzing the Outcome at Follow-Up on Patients (Figure 3)

- it included 12 articles [4,16,18,19,21,23,24,26,33,37,38,40];
- the follow-up time was 16 months ± 25.5 SD on average;
- in eight studies PEIs obtained a superior efficacy on patient cared for outcomes in a statistically significant way compared to control groups without PEIs:
  the study by Perlick et al. [38], which reported a reduction in depression scores on the Hamilton Depression Rating Scale; the study by Rami et al. [26], which reported a statistically significant reduction in psychotic symptoms on the PANSS; the study by Chien et al. [19], which highlighted a reduction in psychotic symptoms on the PANSS; the study by Chien et al. [4], which highlighted a reduction in psychotic symptoms on the PANSS; the study by Peris et al. [37], which underscored a better response rates in the Clinical Global Impression-Improvement scale;
- the study by Rahayu et al. [24], which reported a reduction in prodromal psychosis symptoms in the Prodromal Questionnaire-16; the study by Sepúlveda et al. [18], which reported an improvement in behaviors on the Eating Attitudes Test-26; and the study by Zhang et al. [21], which recorded a better recovery level on the Mental Health Recovery Measure;
- the I^2^ = 88.89% showed the high heterogeneity of the model.

### 3.8. Qualitative Outcomes

In this section, qualitative outcomes for caregivers are described through a narrative synthesis. These outcomes relate to perceived stress, caregiving burden, understanding of psychiatric illness, related dynamics, and vulnerability to developing physical and mental illnesses. For care recipients, the outcomes focus on symptom improvement, relapse rates, and the effectiveness of planned interventions for achieving therapeutic success.

#### 3.8.1. The Following Articles Analyzed Outcomes in Both Caregivers and Patients

- Kopelovich et al. [31]: Using Psychosis REACH (Psychosis Recovery by Enabling Adult Carers at Home), the study found that training can improve the mental health, skills, and relational capacities of families and caregivers. Results indicate that families noticed a reduction in negative caregiving assessments, improved communication, coping strategies, and problem-solving. Care recipients diagnosed with schizophrenia spectrum disorders reported reduced anxiety and depression from pre- to post-training, measured using the HADS (Hospital Anxiety and Depression Scale). The study notes that early family interventions regarding psychosis are recommended by U.S. national guidelines as standard treatment for schizophrenia. It concludes that this recovery-oriented intervention can positively influence both caregivers’ and care recipients’ perceptions of their mental health and interpersonal dynamics.
- Miklowitz et al. [32]: This study discusses pharmacotherapy combined with family-focused therapy (FFT), which includes psychoeducation, communication skills training, and problem-solving for patients and families. It is associated with greater reductions in mood symptom severity and relapse times in patients with bipolar disorder and major depression, compared to standard psychoeducational treatment. Previous research supports these findings, demonstrating the tool’s validity, with greater improvements in positive family processes like cohesion and constructive communication, as well as greater reductions in conflict compared to shorter psychoeducational interventions.
- Wong et al. [25]: This article highlights the caregiver’s perspective, aiming to understand key aspects of managing psychosis cases. The Early Psychosis Intervention Programme (EPIP) results show that caregivers act as “bridges”, collaborating and consulting with other professionals or care providers, especially improving crisis management for their loved ones. For care recipients, acquiring skills to better manage crises and recognizing the need to seek help is a significant step toward improving their recovery journey.

#### 3.8.2. Specific Outcomes in Caregivers

Perceived stress, family cohesion and conflicts, fears of handling crises and difficulties in communicating among caregivers were explored in the following studies:

- Batchelor et al. [12] proposed alternative forms of support for families and caregivers through the use of technology. This study investigated the use of a remote telemedicine intervention, demonstrating that personalized support services combined with interactions with expert caregivers have positive impacts on the well-being and caregiving perspective of the patient. Almost all participants reported a positive experience with COPe-support (Carers for People with Psychosis e-support), advocating for its continued implementation in the future.
- Izon et al. [17], through individual interviews, explored aspects that may facilitate support for individuals at risk of mental health issues. Using individual and family cognitive behavioral therapy (IFCBT), the study highlighted three key aspects: “expectations and knowledge”, “personal factors of the family/caregiver”, and “relational aspects”. The emerging themes include frustration with the mental health service system, feelings of uncertainty, health and well-being issues, work–life balance, access to emotional support services, practical coping strategies, and responsibility for the ill individual. Family members described symptoms of depression and antisocial behaviors as the most challenging to manage as they struggle to empathize with the thoughts leading to such behaviors, which creates distance between them and their loved ones. This, in turn, triggers feelings of guilt, fear, and persistent sadness. The study emphasizes that providing support to families, including psychoeducation, helps explore more appropriate strategies for addressing emerging situations during the caregiving process.
- Lal et al. [29]: This study revealed that caregivers feel anxious and unprepared in handling a crisis episode and recognizing and dealing with potential relapses, and have ineffective coping strategies and limited resources. Additionally, they express an unmet need for communication with the professionals treating their ill relatives. Caregivers report that a crisis episode is traumatic not only for the patient but also for themselves. They have expressed a desire to be better informed about the illness, receive emotional support, and learn coping strategies to prevent relapses. Finally, they wish to be more involved in the care process, starting with having their observations considered.
- Lo et al. [20]: This study introduces the “Photovoice” method, which promotes dialogue about personal experiences through sharing photographs. Researchers suggest that this approach can enhance understanding of how a mindfulness-based family psychoeducation program (MBFPE) can reduce caregiver burden and improve their caregiving experience. The study observed that caregivers learned to use mindfulness to reduce hostility and emotional over-involvement, better regulating strong emotions. The application of “Photovoice” offers an additional approach to increase caregivers’ awareness during the MBFPE psychoeducation process.
- Nolan and Petrakis [27]: This case report discusses the effectiveness of psychoeducational interventions, recommending their implementation because they meet the needs of families. The psychoeducational models used include the stress vulnerability model and the phases of psychosis model. The former provides a simple visual representation of how various stressors contribute to a person’s mental deterioration. Families often seek to understand the causes of the current situation by examining past events in hopes of identifying significant triggers. The nurse found the phases of psychosis model useful in managing both diagnostic uncertainty in early psychosis and caregivers’ guilt, while also discussing early warning signs and offering hope for the future improvement of the patients’ symptoms.
- Pollio et al. [39]: In their study, they examined the impact of patient preferences in a psychoeducational intervention for families, aligning it with a recovery-oriented model. Research indicates that psychoeducational intervention programs are associated with reduced relapse rates, improved recovery, and family well-being by decreasing burden and distress. The most frequently studied topics by Psychoeducation Responsive to Families (PERF) groups include problem-solving, communication, and available community resources. The results presented here support the idea that patients should have greater freedom in defining their educational needs without being excluded from opportunities deemed necessary by professionals. This study concludes by highlighting the potential of integrating psychoeducation and other structured interventions more solidly into a recovery model.
- Sheikhan et al. [30]: This study highlights that caregivers of people with psychiatric disorders have a higher rate of developing mental health problems compared to the general population. The proposed intervention demonstrated increased caregivers’ ability to manage the challenges of their young relatives’ illness. Additionally, participation in such programs positively impacted the intra/interpersonal sphere of the participants. The study recommends implementing the Family Connections (FC) program as an intervention for both young individuals and caregivers.

## 4. Discussion

The studies included in this systematic review analyzed the effectiveness of psychoeducational interventions in improving the psychological well-being and quality of life of both caregivers and their adolescent/young adult patients with mental disorders in different clinical settings.

Various indicators were selected to evaluate the effectiveness of PEIs: short- and long-term changes in patient symptoms and social functioning, reduction in intra-family conflicts, decreases in hospital admissions and increases in adherence to treatment.

In this systematic review, different settings of PEIs were highlighted in the selected articles: individual, group and family sessions, self-administered questionnaires completed remotely or in person, and administration of scales by multiple professionals, caregivers or expert patients.

All the selected studies reported PEIs on caregivers according to the inclusion criteria, but some of them focused on the effectiveness only on physical and mental health, stress and quality of life of caregivers, others on symptomatic improvement of caregiver recipients, and others on both caregivers and recipients. The included studies, although presenting similar results, are characterized by extreme heterogeneity in the psychometric scales administered, in the follow-up periods of the studies and in the sample sizes, which reduced the validity of our meta-analyses.

We reported that “Parent” was the most frequent category of caregivers, and “Schizophrenia Spectrum Disorder” was the most frequent psychiatric disorder suffered by recipients. The ages of the assisted persons varied from a minimum of 11 years to a maximum of 25 years, identifying a child–adolescent population of recipients. The age range of caregivers was broad, from 18 to 85 years, suggesting a heterogeneous sample based on age.

The psychometric scales relating to care burden, care experience and caregiver development of problem-solving skills showed significant improvement in the groups of caregivers treated with family psychoeducation interventions compared to others [4,37,38]. Katsuki et al.’s [22] study found a near-zero rate of caregiver dropout from family sessions, with 84% of participants who adhered to treatment, suggesting full satisfaction with the PEIs implemented. All included studies, except the study by Beck et al. [16], highlighted that caregivers treated with PEIs aimed at improving caregivers’ quality of life and family cohesion reported significant improvement compared to controls. In particular, the studies that analyzed the effect of psychoeducation in both caregivers and people cared for, underscored improvement in the caregivers’ ability to manage psychotic disorders in the persons cared for and better knowledge of disorders [23,25].

In many of the qualitative studies included, PEIs were shown to be effective in improving caregivers’ global psychological health, reducing their negative reactions to patients’ symptoms and increasing their emotional well-being and awareness of personal resources, with a concomitant reduction in family conflicts and psychological distress [12,17,27,29]. Other aspects appreciated by caregivers were represented by the increased knowledge and understanding of the recipient’s disorder, with the possibility of improving crisis management and applying appropriate coping strategies and a concomitant improvement in the quality of life of both caregiver and patient [23,25,31,32].

The selected studies report improvements in the quality of life of patients, with greater family cohesion after the psychoeducational interventions [1,37] and indirect improvements in the symptoms of patients cared for by caregivers. In the studies in which the symptomatology of patients with psychosis was analyzed, a reduction in psychotic symptoms, an improvement in social functioning and greater adherence to pharmacological therapy were reported [4,19,26]. Moreover, PEIs focused on family reduced the rate of patient hospitalization. The effect of PEIs on the depression symptoms of assisted patients varied depending on the kind of intervention applied: the study by Rinne et al. [40] reported a reduction in the recurrence of depressive symptoms and an increase in self-esteem both in the group treated with family-focused therapy and in the one treated with standard psychoeducation. In the study by Bernal et al. [28], implemented on patients with major depression, the group with PEIs did not improve scores on the psychometric scales of depression, but patients reported a better adjustment to the family. In the study of Peris et al. [37], symptomatic improvement and an increase in remission rates in patients with obsessive-compulsive disorder were highlighted, whereas in the study by Sepúlveda et al. [18], improvements in behaviors associated with eating disorders and subjective well-being were observed.

We applied two meta-analyses for separately analyzing the outcomes on caregivers and on patients in the studies included. Despite the extreme heterogeneity with I^2^ approximately or above 90% related to both the PEIs that were implemented and the psychometric scales used, we reported in both meta-analyses of caregiver and patient studies statistically significant superior efficacy of PEIs compared to the control groups. These results suggest that PEIs had a positive effect on improving caregivers’ well-being, the mental health conditions of their adolescent and young patients with mental disorders, and family functioning. Therefore, it should be emphasized that PEIs for caregivers indirectly promote the improvement of the person being cared for with a positive impact on the entire family. These results suggest that PEIs had a positive effect on improving caregivers’ well-being, the mental health conditions of their adolescent and young patients with mental disorders, and family functioning. Therefore, it should be emphasized that PEIs for caregivers indirectly promote the improvement of the person being cared for, with a positive impact on the entire family, especially if the caregiver is one of the parents of the patient being cared for, as highlighted by most of the selected studies.

Nevertheless, we have to put highlight the evidence that family psychoeducation alone would not be successful. However, in addition to prescription drugs and other psychological and social treatments, psychoeducation could make a significant difference in outcomes [43].

The clinical implications highlighted by this review are both for the patient’s treatment, mainly represented by the positive impacts on treatment adherence and outcome, and for the caregiver’s quality of life [44,45]. In light of our results, we can hypothesize that a more widespread implementation of family psychoeducation in all psychiatric settings could reduce both treatment costs and patient dropouts as well as improving relationships within patients’ families. Future studies could highlight which elements of family psychoeducation correlate with improvements in patient’s treatment outcomes and which with improvements in caregivers’ quality of life.

### Strengths and Limitations

A strength of this study is represented by the use of multiple sources for collecting studies and systematic and structured methodology. In order to collect the greatest number of articles, the search was carried out on five different databases and no limits were set relating to the geographical origin of the studies. Furthermore, no exclusion criteria were defined regarding the type of data and results investigated, including studies with both quantitative and qualitative outcomes.

The limitations concern first of all the study language, with the search strategy built considering only articles in English. Another limitation is the heterogeneity of the included studies due to different psychometric scales, sample sizes and follow-up periods. The limited number of studies was not sufficient to allow us to perform a stratification analysis. Our systematic review was not able to highlight whether a certain type of psychoeducational intervention may be more effective than another, reporting similar effects for therapeutic assistance rehabilitation treatments with different theoretical constructs.

## 5. Conclusions

Our review shows that psychoeducation can be an effective intervention in improving outcomes for assisted people, reducing the symptoms, hospitalizations and stress related to the disease, and concomitantly favoring an improvement in quality of life in both caregivers and recipients. Regarding caregiver burden, our review did not report exhaustive results due to the heterogeneity of the selected studies but suggested that PEIs generally improve quality of life and the perception of burden in caregivers. It should also be emphasized that the effectiveness of psychoeducation may vary depending on the severity of the psychological conditions of people cared for, the duration and method of delivery as well as adherence to intervention. Therefore, it is necessary to consider various factors related to the clinical and care characteristics of the assisted patient and caregiver and to the setting in which PEI is implemented.

Our analysis highlights that a parent, particularly the mother, was the most frequent caregiver in cases of mental disorders in adolescents or young adults, which suggests the burden and level of involvement that these disorders can place on families. It is no coincidence that our review highlights that caregiver interventions are able to reduce family conflicts, promoting family cohesion.

In light of these results, we confirm that PEIs for caregivers can not only reduce emotional burden, but above all can positively influence the course of the disorder of the person being cared for, and the functioning of the patient’s family, due to the close relationship between mental disorders and the living environment.

## Figures and Tables

**Figure 2:**
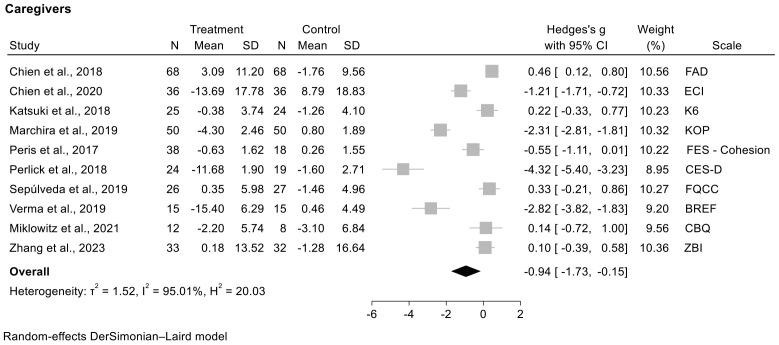
Forest plot with meta-analysis of studies analyzing effectiveness of psychoeducation interventions in caregivers [1,4,18,19,21,22,23,33,37,38]. The squares represent mean differences, and horizontal lines represent their 95% CI. The area of each square is proportional to the weight of the study in the meta-analysis. The diamonds represent the combined standardized mean.

**Figure 3:**
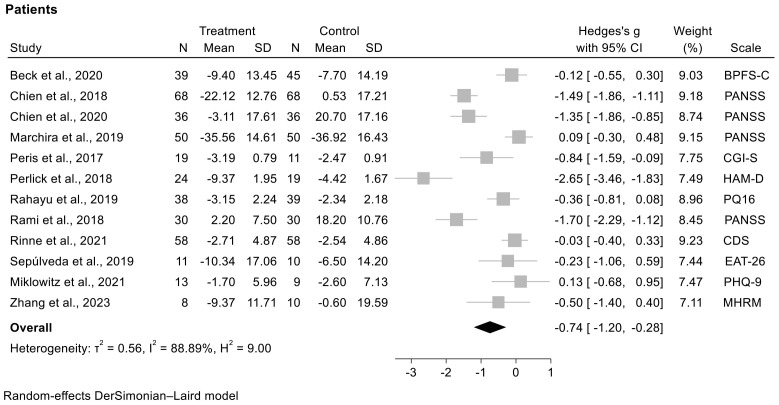
Forest plot with meta-analysis of studies analyzing effectiveness of psychoeducation interventions in patients [4,16,18,19,21,23,24,26,33,37,38,40]. The squares represent mean differences, and horizontal lines represent their 95% CI. The area of each square is proportional to the weight of the study in the meta-analysis. The diamonds represent the combined standardized mean.

**Table 2:** Characteristics of the selected studies with outcomes only for caregivers.

Authors, Year of Publication	Setting and Follow-Up	Caregiver Intervention	Professionals	Primary Caregiver Outcome
Batchelor et al., 2022 [12]	Web intervention 8 months	Online psychoeducational support	Mental health nurses	Flexible and personalized remote support and engagement with colleagues and experts
Izon et al., 2020 [17]	Individual sessions 6 months	Individual and family cognitive behavioral therapy	Not reported	Improvement in relational expectations and coping strategies clinically evaluated
Lal et al., 2019 [29]	Group sessions Follow-up not reported	Early psychosis intervention program	Families	Qualitative analysis of caregiver concerns
Lo et al., 2022 [20]	Outpatients Follow-up not reported	Mindfulness-based family psychoeducation program (MBFPE) combined with the “Photovoice” method.	Qualified instructors experienced in mindfulness	Caregivers learned to use mindfulness methods to reduce their hostility and excessive emotional involvement, better regulating strong emotions
Nolan and Petrakis 2019 [27]	Inpatients Follow-up not reported	Psychoeducation on stress vulnerability phases of psychosis	Senior mental health nurses	The stress vulnerability and phases of psychosis models are effective educational tools for caregivers
Pollio et al., 2017 [39]	Outpatients 12 months	Family psychoeducation	Psychiatrists, psychiatric nurses, and social workers	Topics of highest priority are family life and independence
Sheikhan et al., 2021 [30]	Outpatients 3 months	Support and training program for caregivers	Trained family members of psychiatric patients and facilitator	Improvement in mental health awareness and. management of family members
Verma et al., 2019 [1]	Outpatients 6 months	Family Psychoeducation	Not reported	Improved quality of life indicators on the WHOQOL-BREF: experimental group baseline = 36.47 ± 5.82 SD at outcome = 51.87 ± 6.67 SD; control baseline = 36.47 ± 5.82 SD at outcome = 32.27 ± 5.06 SD
Weintraub et al., 2021 [42]	Outpatients 48 months	Family-focused therapy (FFT) or standard psychoeducation	Not reported	Reduction in maternal stress level on the SCL-90: decreases on average by 0.41 at each 4-month follow-up; FFT improves family cohesion (FACES-II) and maternal stress levels

FACES—Family Adaptability Cohesion Evaluation Scale; FFT—Family-Focused Therapy; SCL-90—Symptom Check List; SD—Standard Deviation; WHOQOL-BREF—World Health Organization Quality of Life-BREF.

**Table 3:** Characteristics of the selected studies with outcomes only on recipients.

Authors, Year of Publication	Setting and Follow-Up	Caregiver Intervention	Professionals	Primary Patient Outcome
Beck et al., 2020 [16]	Outpatients 75 months	Mentalization-based group therapy	Nurses, psychologists, social workers, psychiatrists	BPFS-C score showed no statistically significant difference between the treated group and the control group at the end of follow-up
Miklowitz et al., 2022 [34]	Outpatients 48 months	Family-focused therapy (FFT) or enhanced usual care (brief family psychoeducation and individual support)	Researchers	FFT in combination with pharmacotherapy was associated with longer periods free from mood episodes and greater reductions in suicidal ideation and behavior among young individuals at high risk for bipolar disorder
O’Donnell et al., 2017 [35]	Outpatients 24 months	Family-focused treatment for adolescents—EC	Not reported	Improvements in quality of life on the KINDL questionnaire in the dimensions of physical well-being and friendship skills
O’Donnell et al., 2020 [36]	Outpatients 24 months	Family-focused treatment	Not reported	Improvement in family cohesion, adaptability, and reduction in conflicts in the treated group (FACES-II)
Rahayu et al., 2019 [24]	Outpatients 2 months	Cognitive therapy and family psychoeducation	Therapists and nurses	Reduction in prodromal psychosis symptoms (PQ16: reduction from 947 to 632 in the treated group; *p* = 0.00) and increase in self-esteem (RSE: increase from 1387 to 2239; *p* = 0.00)
Rami et al., 2018 [26]	Outpatients 6 months	Behavioral family psychoeducational program	Researchers	Reduction in psychotic symptoms on the PANSS (t = 7.3; *p* < 0.001), improvement in social functioning on the SFQ (t = −7.9; *p* < 0.001), quality of life on the QOLS (t = −6.9; *p* < 0.001) and attitude towards medications on the DAI (t = −7.6; *p* < 0.001), with a statistically significant difference between the treated group and the control group
Rinne et al., 2021 [40]	Outpatients 24 months	Family-focused therapy for individuals at high clinical risk or Psychoeducation	Researchers	Depression score decreased on the CDS but independently of the type of treatment administered (family-focused therapy or psychoeducation)

BPFS-C—Borderline Personality Features Scale for Children-11; CDS—Children’s Depression Scale; DAI—Drug Attitude Inventory; FACES—Family Adaptability Cohesion Evaluation Scale; EC—enhanced care; FFT—Family-Focused Therapy; QOLS—Quality of Life Scale, PANSS—Positive and Negative Syndrome Scale; PQ-16—Prodromal Questionnaire 16-item version; RSE—Rosenberg Self-Esteem Scale; SFQ—Social Functioning Questionnaire.

**Table 4:** Characteristics of the selected studies with outcomes of both caregivers and recipients.

Authors, Year of Publication	Setting and Follow-Up	Caregivers Intervention	Professionals	Outcomes	
				Caregivers	Patients
Bernal et al., 2019 [28]	Outpatient 12 months	Psychological education laboratories	Clinical psychologists	Although the treatment did not optimize depression scores, parents gave positive feedback on their parenting styles	The treated group perceived a reduction in family system maladjustment on the FES while the control group perceived an increase (effect size of 1.51 on the curve)
Chien et al., 2018 [19]	Outpatient 48 months	Family support groups and psychoeducation	Caregiver trainer and psychiatric nurse	Improvement of family functioning on the FAD at the end of follow-up	Reduced hospitalization rate and psychotic symptoms on the PANSS at the end of follow-up
Chien et al., 2020 [4]	Outpatient 6 months	Family psychoeducation	Family caregiver facilitator and psychiatric nurse	Improvement in caregiving experience on the ECI	Reduction in psychotic symptoms on the PANSS
Katsuki et al., 2018 [22]	Clinical 8 months	Brief multifamily psychoeducation (BMP) and counselling	Psychotherapist and nurses	Reduction in psychological stress on the K6 but no statistically significant benefit of BMP intervention	FAD scores reduced in the intervention group
Kopelovich et al., 2021 [31]	Outpatient–inpatient 4 months	Psychosis REACH: intervention aimed at psychosis recovery at home by enabling caregivers	Authors	Reduction in negative care assessments on the ECI: positive total score in post-training follow-up	Reduction in hospital anxiety and depression on the HADS
Marchira et al., 2019 [11]	Group sessions 6 months	Brief psychoeducation	Not reported	Improvement in psychosis knowledge on the KOP	Reduction in psychotic symptoms on the PANSS not statistically significant at 6-month follow-up
Miklowitz et al., 2020 [32]	Outpatient 4 months	Family-focused therapy—EC (psychoeducation)	Not reported	Reduced vulnerability to bipolar disorder evaluated on the FFT scale	Unchanged scores on the SIQ in treated and control group
Miklowitz et al., 2021 [33]	Group sessions 4 months	High–low Intensity training of family-focused therapy	Healthcare workers	Decrease in family conflicts evaluated on the CBQ	No significant change in patient health on the PHQ-9 in treated and control group
Peris et al., 2017 [37]	Outpatient 3 months	Positive family interaction therapy	Clinical psychologists and psychology PhD students	Reduction in conflicts on the FES and improvement in family cohesion	Improved response rates on the CGI-I scale (68% treated group vs. 40% control group)
Perlick et al., 2018 [38]	Outpatient 6 months	Family-focused treatment adapted to caregiver only	Therapists	Improvement in overall psychological health and reduction in depression symptoms	Reduction in depression symptoms on the HAM-D score from 15.22 to 5.85 in the treated group and from 14.53 to 10.11 in the control group score reduced
Sepúlveda et al., 2019 [18]	Outpatient 6 months	Skill-based workshop (SBW) or psychoeducation (PE)	Researchers	Reduction in negative reactions to illness on the FQ and improvement in acceptance of symptoms on the AESED in both groups	Improvement in eating disorder-associated behaviors in the PE group
Weintraub et al., 2019 [41]	Outpatient 24 months	Family-focused therapy (FFT) or brief psychoeducation	Not reported	Reduction in family conflicts on the CBQ in families of patients with bipolar disorder and ADHD comorbidity	Reduction in manic symptoms in patients with bipolar disorder and ADHD comorbidity on the PSR: 18% reduction in the FFT group compared to 2% in the control group
Wong et al., 2019 [25]	Outpatient 12 months	Focus groups discussions	Team of psychiatrists and case managers	Improvement in crisis management	Improvement in crisis management
Zhang et al., 2023 [21]	Outpatient 9 months	Mindfulness-based family psychoeducation (MBFPE) program or ordinary family psychoeducation (FPE) program	Clinicians	Slight worsening in the primary outcome score: caregivers’ burden on the ZBI	Better recovery levels recorded with the MHRM after MBFPE compared to FPE (but not statistically significant)

ADHD—Attention Deficit Hyperactivity Disorder; AESED—Accommodation and Enabling Scale for Eating Disorder; BMP—Brief Multifamily Psychoeducation; CBQ—Children’s Behavior Questionnaire; CES-D—Center for Epidemiologic Studies Depression Scale; CGI-I—Clinical Global Impression-Improvement; EAT-26—Eating Attitude Test-26; ECI—Emotional Competence Inventory; FAD—Family Assessment Device; FFT—Family-Focused Therapy; FES—Family Environment Scale; HADS—Hospital Anxiety and Depression Scale; HAM-D—Hamilton Rating Scale for Depression; K6—Kessler-6 Psychological Distress Scale; KOP—Kogan’s Attitudes toward Older People; MHRM—Mental Health Recovery Measure; PANSS—Positive and Negative Syndrome Scale; PHQ-9—Patient Health Questionnaire-9; PSR—Psychiatric Rating Scale; REACH—Raising Early Awareness and Creating Hope; SIQ—Suicide Ideation Questionnaire; SD—Standard Deviation; ZBI—Zarit Burden Inventory.

**Table 5:** The Revised Cochrane Risk of Bias Tool of selected studies.

Study	Randomization Process	Effect of Assignment to Intervention	Missing Outcome Data	Measurement of the Outcome	Selection of the Reported Result	Overall Risk of Bias
Beck et al., 2020 [16]	Some concerns	Some concerns	Low	Low	Low	Some concerns
Bernal et al., 2019 [28]	Some concerns	Some concerns	Some concerns	Low	Some concerns	Some concerns
Chien et al., 2018 [19]	Low	Some concerns	Low	Low	Low	Low
Chien et al., 2020 [4]	Low	Some concerns	Low	Low	Low	Low
Katsuki et al., 2018 [22]	Low	Some concerns	Low	Low	Low	Low
Marchira et al., 2019 [23]	Low	Some concerns	Some concerns	Low	Low	Some concerns
Miklowitz et al., 2020 [32]	Low	Some concerns	Some concerns	Low	Low	Some concerns
Miklowitz et al., 2021 [33]	Low	Low	Low	Low	Low	Low
Miklowitz et al., 2022 [34]	Low	Some concerns	Low	Low	Some concerns	Some concerns
O’Donnell et al., 2017 [35]	Low	Low	Some concerns	Low	Some concerns	Low
O’Donnell et al., 2020 [36]	Low	Low	Some concerns	Low	Some concerns	Low
Peris et al., 2017 [37]	Low	Low	Low	Low	Low	Low
Perlick et al., 2018 [38]	Some concerns	Some concerns	Low	Low	Low	Some concerns
Rahayu et al., 2019 [24]	Some concerns	Some concerns	Low	Low	Some concerns	Some concerns
Rami et al., 2018 [26]	Low	Some concerns	Low	Low	Low	Low
Rinne et al., 2021 [40]	Low	Some concerns	Low	Low	Low	Low
Sepúlveda et al., 2019 [18]	Low	Some concerns	Some concerns	Low	Low	Some concerns
Verma et al., 2019 [1]	Low	Some concerns	Low	Low	Low	Low
Weintraub et al., 2019 [41]	Low	Low	Some concerns	Low	Some concerns	Low
Weintraub et al., 2021 [42]	Low	Low	Some concerns	Low	Some concerns	Low
Zhang et al., 2023 [21]	Low	Some concerns	Low	Low	Low	Low
